# Weight Loss Instead of Weight Gain within the Guidelines in Obese Women during Pregnancy: A Systematic Review and Meta-Analyses of Maternal and Infant Outcomes

**DOI:** 10.1371/journal.pone.0132650

**Published:** 2015-07-21

**Authors:** Mufiza Zia Kapadia, Christina K. Park, Joseph Beyene, Lucy Giglia, Cindy Maxwell, Sarah D. McDonald

**Affiliations:** 1 Department of Obstetrics and Gynecology, McMaster University, Hamilton, Ontario, Canada; 2 Department of Clinical Epidemiology and Biostatistics, McMaster University, Hamilton, Ontario, Canada; 3 Department of Pediatrics, Division of General Pediatrics, McMaster University, Hamilton, Ontario, Canada; 4 Department of Obstetrics and Gynaecology, Division of Maternal Fetal Medicine, Mount Sinai Hospital and University Health Network, University of Toronto, Toronto, Ontario, Canada; 5 Department of Obstetrics and Gynecology, Division of Maternal Fetal Medicine, McMaster University, Hamilton, Ontario, Canada; 6 Department of Radiology, McMaster University, Hamilton, Ontario, Canada; The University of Kansas Medical Center, UNITED STATES

## Abstract

**Background:**

Controversy exists about how much, if any, weight obese pregnant women should gain. While the revised Institute of Medicine guidelines on gestational weight gain (GWG) in 2009 recommended a weight gain of 5–9 kg for obese pregnant women, many studies suggested even gestational weight loss (GWL) for obese women.

**Objectives:**

A systematic review was conducted to summarize pregnancy outcomes in obese women with GWL compared to GWG within the 2009 Institute of Medicine guidelines (5–9 kg).

**Design:**

Five databases were searched from 1 January 2009 to 31 July 2014. The Cochrane Handbook for Systematic Reviews of Interventions and the PRISMA Statement were followed. A modified version of the Newcastle-Ottawa scale was used to assess individual study quality. Small for gestational age (SGA), large for gestational age (LGA) and preterm birth were our primary outcomes.

**Results:**

Six cohort studies were included, none of which assessed preterm birth. Compared to GWG within the guidelines, women with GWL had higher odds of SGA <10^th^ percentile (adjusted odds ratio [AOR] 1.76; 95% confidence interval [CI] 1.45–2.14) and SGA <3^rd^ percentile (AOR 1.62; 95% CI 1.19–2.20) but lower odds of LGA >90^th^ percentile (AOR 0.57; 95% CI 0.52–0.62). There was a trend towards a graded relationship between SGA <10^th^ percentile and each of three obesity classes (I: AOR 1.73; 95% CI 1.53–1.97; II: AOR 1.63; 95% CI 1.44–1.85 and III: AOR 1.39; 95% CI 1.17–1.66, respectively).

**Conclusion:**

Despite decreased odds of LGA, increased odds of SGA and a lack of information on preterm birth indicate that GWL should not be advocated in general for obese women.

## Introduction

Obesity is a global epidemic affecting an estimated 500 million people [1]. Among women of childbearing age, up to three in ten are obese [2,3], defined as having a body mass index (BMI) greater than or equal to 30 kg/m^2^, 10% of whom meet the criteria for obesity class II (BMI: 35–39.9 kg/m^2^) or III (BMI: ≥ 40 kg/m^2^) [3]. Pre-pregnancy obesity has been associated with both short and long term pregnancy complications for the mother [4–7] and their offspring [8–13], hence minimizing adverse outcomes in this high-risk group is a public health priority.

Controversy exists about the amount of how much, if any, weight obese pregnant women should gain [14–17]. The gestational weight gain (GWG) guidelines were recently revised by the Institute of Medicine (IOM) in 2009, and recommended a weight gain of 5–9 kg for *all classes* of obesity [18]. There were a lack of sufficient evidence to make specific recommendations for each of the different obesity classes [18]. Since adverse pregnancy complications are often more frequently associated with more severe obesity such as class II and/or III [5,6,8,19], the question has arisen whether the same weight gain requirements should apply to all classes of obese women. In another systematic review, we found increased odds of small for gestational age (SGA) and preterm birth, but decreased odds of large for gestational age (LGA), for obese women with positive weight gain below the guidelines (no weight loss) compared to those with weight gain within the guidelines [20].

Although gestational weight loss (GWL) during pregnancy is not recommended by the IOM, about 8% of all pregnant women reported *attempting* to lose weight, with the highest prevalence (13%) reported in obese women [21]. Moreover, the prevalence of *actual* GWL increases with increasing obesity class, reaching as high as 15% in obesity class III [22,23].

Recent evidence has examined lower weight gain in obese women than what is currently recommended by the IOM guidelines. Margerison-Zilko et al. suggested a weight gain of <5 kg for obese women to yield 10% probabilities of SGA and LGA with reduced risk of cesarean section, postpartum weight retention and child overweight [16]. Oken et al. identified that the lowest prevalence of five adverse outcomes (preterm birth, SGA, LGA, postpartum weight retention and child obesity at 3 years) were achieved with a GWL of 7.6 kg in obese women [15]. Beyerlein et al. suggested that GWL is beneficial only in non-smoking women to yield a 20% joint predicted probability of SGA and LGA [14]. Moreover, the uncertainty surrounding the current IOM guidelines is reflected in the American College of Obstetricians and Gynecologists Committee’s recent opinion paper stating that "for an obese pregnant woman who is gaining less weight than recommended but has an appropriately growing fetus, no evidence exists that encouraging increased weight gain to conform with the updated IOM guidelines will improve maternal and fetal outcomes” [17].

A recent Cochrane systematic review of randomised controlled trials (RCT) of GWL in obese women failed to identify any interventional studies [24] and noted a need for evidence about whether weight loss is safe. Hence, we responded to the call for additional evidence about outcomes with GWL, using the highest evidence available by including observational studies to examine adverse pregnancy outcomes in obese women overall, and in each of the three obesity classes. Therefore, the aim of this systematic review was to present a systematic, unbiased quantitative summary of the evidence from RCTs and observational studies examining the association between adverse pregnancy outcomes in singleton gestations in obese women (overall and within each obesity class) with GWL compared to GWG within the guidelines.

## Materials and Methods

The criteria outlined in the Cochrane Handbook for Systematic Reviews of Interventions [25] and the PRISMA Statement were followed (S1 Table) [26]. Similar methodology was employed as in the previous meta-analysis examining gestational weight gain less than that recommended in the guidelines, but still positive [20].

### Search Strategy

The following databases were searched between January 1, 2009 and July 31, 2014: Medline, Embase, Cochrane Register, CINAHL and Web of Science. This time frame was selected in accordance with the release of the new 2009 IOM guidelines [18]. Specific search strategies were developed for each database in consultation with a librarian who had expertise in health sciences systematic reviews (S1 File). All included studies were searched in their reference lists for potential articles for inclusion. Bibliographic software (Endnote version X6, CA, USA) was used to catalog all citations and discard duplicates.

### Eligibility Criteria

Studies were required to compare obese pregnant women with GWL (<0 kg) and those with GWG within the 2009 IOM guidelines (5–9 kg) to be included, investigated in obesity overall (BMI ≥ 30 kg/m^2^), and/or in any class of obesity (I: BMI 30–34.9 kg/m^2^, II: BMI 35–39.9 kg/m^2^ and III: BMI ≥ 40 kg/m^2^). RCTs, and cohort, case-control and cross-sectional studies were eligible study designs. Studies were included if they reported on singleton pregnancies, since outcomes are markedly different in twins, and were published in English. Ineligible study designs were conference proceedings reported only as abstracts, editorials, opinions, and review articles, as were duplicate or secondary publications.

### Outcome Measures

Primary outcomes under study were SGA (less than 10^th^ percentile of birth weight for sex and gestational age), LGA (greater than 90^th^ percentile of birth weight for sex and gestational age) and preterm birth (less than 37 weeks [inclusive of <32 weeks and 32–36 weeks], less than 32 weeks, or between 32 and 36 weeks). These three outcomes were selected due to the critical importance of maternal weight gain on the neonatal growth and potentially pregnancy duration as per the IOM, and the resultant neonatal mortality and morbidity [27,28].

Secondary outcomes regarding infants included other definitions of SGA (less than the 3rd and 5th percentile), other measures of low birth weight (low birth weight defined as less than 2500 g, very low birth weight defined as less than 1500 g, extremely low birth weight defined as less than 1000 g), other definitions of LGA (greater than the 95th and 97th percentile), other measures of high birth weight (macrosomia defined as greater than 4000 g or 4500 g), shoulder dystocia, severe neonatal morbidity (e.g. Apgar score less than 7 at 5 minutes, congenital malformation, intraventricular haemorrhage, low arterial cord blood pH, neonatal hypoglycemia, neonatal intensive care unit admission, necrotising enterocolitis, newborn resuscitation, respiratory distress syndrome, retinopathy of prematurity, or transient tachypnea of the newborn), and perinatal mortality (fetal death and early neonatal mortality).

Secondary outcomes pertaining to mothers were pre-eclampsia or pregnancy-induced hypertension, gestational diabetes mellitus, chorioamnionitis, placenta previa, placenta abruptio, premature rupture of membranes (less than 37 weeks of gestation in the absence of labor), induction of labor, cephalopelvic disproportion, modes of delivery (cesarean birth, operative vaginal delivery through forceps or vacuum), antepartum or postpartum hemorrhage, initiation of breastfeeding, postpartum weight retention up to one year after birth and increase in obesity class postpartum.

### Study Selection

Titles and abstracts of all citations identified in the search were independently assessed by two reviewers (MZK and CKP) for potential study inclusion. If either reviewer considered the citation potentially relevant, the full-text article was retrieved for further independent evaluation, and if not, the reason(s) for study exclusion was documented. An un-weighted kappa statistic was used to assess inter-reviewer agreement for decision for reviewing full text based on titles and abstracts. Uncertainties or disagreements were discussed and consensus was reached, and if unresolved, an independent adjudicator (SDM) was consulted.

#### Assessment of Risk of Bias

The methodological quality of studies were determined using the Cochrane collaboration tool for randomised controlled trials [25], and a modified version of Newcastle-Ottawa Scale [29] with a maximum of seven points for observational studies. ‘Selection,’ ‘Comparability,’ and ‘Outcome’ were the three categories included in the Newcastle-Ottawa Scale for cohort studies. One item under the ‘Selection’ category was excluded (‘demonstration that outcome of interest was not present at start of study’), as the outcomes of interest in our systematic review could not have been present at the start of the included studies and could only be present after giving birth. Therefore, the ‘Selection’ category was modified to award a maximum of three instead four points. Similarly, another item under ‘Outcome’ (‘was follow-up long enough for an outcome to occur’) was deleted, as follow-up until the end of pregnancy was necessary for study inclusion, resulting in a maximum of two instead of three points. The selection of the two 'most important confounders' was based on *a priori* knowledge of their association with gestational weight changes and each outcome [30], awarding a maximum of two points under the ‘Comparability’ category. No validation studies that suggest a cut-off score for rating “low” quality studies are available; hence an arbitrary cut-off of ≤4 was chosen. Quality assessment was independently untaken for each of the included study by two reviewers (MZK and CKP) and disagreements were resolved by process described above.

Each study was assessed for adequate power by determining if the multivariable regression models had a minimum of 10 events per variable for the primary outcome [31].

#### Data Abstraction

Study information on the country of origin, years of study, study design, study setting, participants, inclusion/exclusion criteria, study outcomes, potential confounders; quality assessment; exposure; and the definition of obesity and obesity categories were independently documented by two reviewers (MZK and CKP) from studies using a piloted data extraction form. If presented in the studies, both the number of events and observations, as well as their respective effect estimates (e.g. odds ratio, relative risk) and confidence intervals, were extracted for each outcome. Discrepancies in data extraction were resolved by referring to the source study, and consensus was reached in the same process as in previous steps.

### Data Synthesis

Statistical analyses were performed with Review Manager (Version 5.1; the Cochrane Collaboration, Oxford, England). Meta-analyses for each outcome were undertaken using random effects model [32] since heterogeneity among studies was expected, with weighting of the studies was based on the generic inverse variance method. Meta-analyses were performed for overall effect of all obesity classes and where possible, stratified by obesity class. Effect estimates were pooled to obtain an overall estimate when data were only available for the individual obesity classes [23,33–36], or stratified according to the degree of GWL [33,35] or parity [36]. Unadjusted and adjusted available data were separately pooled, reported as the OR with 95% confidence intervals (CIs) with statistical significance defined as p < 0.05, but multivariable pooled data were preferentially reported to understand the independent effect of GWL (univariate analyses are presented in Supporting Information).

Heterogeneity among studies was evaluated with a Chi-squared test and quantified by using I^2^ statistics, which represents an estimation of the total variation across studies beyond chance [37], such that greater I^2^ values indicate greater heterogeneity between studies. I^2^ values of 25%, 50% and 75% were respectively considered indices of low, moderate and high degrees of heterogeneity [37]. Funnel plots were generated for each outcome when five or more strata or studies were available to assess publication bias. A post-hoc sensitivity analysis was performed for overall obesity examining the effect of a study that reported 99% CI on the primary outcomes [34].

## Results

### Literature Search

**Fig 1:**
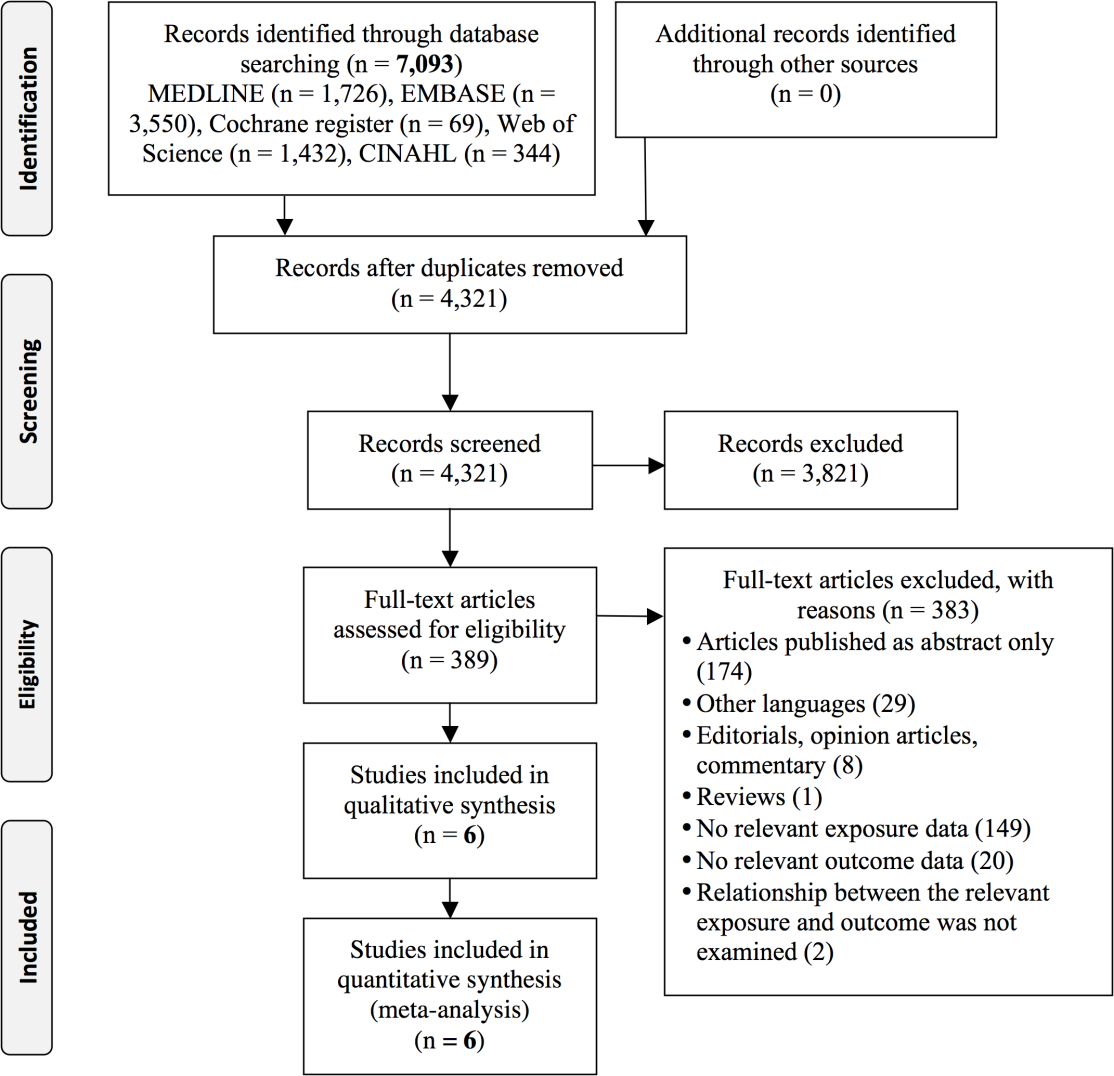
Flow diagram of study selection process.

The electronic searches from the 5 databases revealed 7,093 potentially eligible citations (Fig 1). Of the 4,321 non-duplicate titles and abstracts screened, 389 citations were retrieved for full text review. Six retrospective cohort studies including a total of 16 relevant outcomes were included based on eligibility criteria [23,33–36,38]. No further studies were identified from reviewing the bibliographies of the included studies. There was perfect agreement (kappa = 1) between the reviewers regarding the inclusion of studies. Studies were frequently excluded because they did not categorize participants by pre-pregnancy BMI.

### Study Characteristics

The included studies reported on at least 60,913 obese pregnant women (the number of obese women could not be ascertained in one study [35]). Five studies were American [33–36,38] and one was Swedish [23]. All but one study [38] investigated outcomes according to the three obesity classes. In addition, one study also investigated outcomes for overall obesity [34]. Other information on the study setting and period, exclusion and inclusion criteria, sample size and covariates are presented in Table 1.

**Table 1:** Characteristics of included cohort studies in systematic review of gestational weight loss in obese women and adverse pregnancy outcomes.

Study ID^a^	Study period (years)	Sample size	Study setting	Eligibility Criteria	Confounders	Outcome measures
Blomberg 2011 [23]	1993 to 2008	15,392	Medical Birth Registry, Sweden	**Inclusion criteria**: women with pre-pregnancy BMI ≥ 30 kg/m^2^ and available data on gestational weight gain; included those with diabetes.	**Adjusted for:** Maternal age, parity, smoking.	**Primary**: SGA, LGA Secondary: APGAR score, fetal distress, operative vaginal delivery, caesarean delivery, intra-partum hemorrhage, pre-eclampsia.
Durie 2011 [34]	2004 to 2008	3,765	Five Lakes Region Perinatal Data System, New York (State), USA	**Inclusion criteria:** women with singleton live born neonate ≥ 2 weeks of gestation. **Exclusion criteria:** missing anthropometric measures, extreme BMI, biologically implausible gestational weight gain values.	**Adjusted for:** Chronic diabetes, chronic hypertension, education, maternal age, parity, race/ethnicity, smoking, prior caesarean delivery (only for caesarean delivery).	**Primary**: SGA, LGA. **Secondary**: NICU admission, induction of labor, GDM, caesarean delivery.
Hinkle 2010 [33]	2004 to 2006	36,359	Low income women part of a federally-funded maternal and child health program, (primarily the WIC program), 6 unspecified States, USA	**Inclusion criteria**: maternal pre-pregnancy BMI ≥30 kg/m^2^; non-Hispanic white, non-Hispanic black, and Hispanic with available data from a prenatal and postpartum visit. **Exclusion criteria**: women with missing and implausible data.	**Adjusted for**: Education, gestational age, infant sex, marital status, maternal height, race/ethnicity, smoking. **Excluded:** American Indian and Asian women	**Primary**: SGA, LGA. **Secondary**: Macrosomia.
Kominiarek 2013 [36]	2002 to 2008	4,795	12 institutions/ 19 hospitals), 9 ACOG districts, USA	**Inclusion criteria**: maternal pre-pregnancy BMI ≥ 30 kg/m^2^ and known gestational weight change in a singleton, term (≥37 weeks), live born gestation. **Exclusion criteria**: gestational weight gain outside the range of -20 kg to 50 kg; only the first pregnancy was included if more than one pregnancies within the study period.	**Adjusted for:** Gestational age, insurance, marital status, maternal age, parity, race/ethnicity, smoking.	**Primary**: SGA, LGA Secondary: NICU admission, LBW, shoulder dystocia, APGAR score, operative vaginal delivery, caesarean delivery.
Park 2011 [35]	2004 to 2007	NR	Florida live-birth certificates, USA	**Inclusion criteria**: women aged 18–40 years with a singleton full-term live birth (37–41 weeks of gestation); available information for pre-pregnancy BMI, gestational weight change, and outcomes.	**Adjusted for:** Education, gestational age, infant birth year, infant sex, maternal age, number of prenatal visits, parity, race/ethnicity, smoking, WIC program participation. **Excluded:** Women with chronic diabetes or hypertension.	**Primary**: SGA, LGA.
Vesco 2011 [38]	2000 to 2005	602	Kaiser Permanente Northwest electronic medical records, Oregon and Washington states, USA	**Inclusion criteria**: singleton pregnancies with live births ≥ 37 weeks gestation who delivered within Kaiser Permanente Northwest not-for-profit health organisation with measured maternal weight between 6 months before pregnancy and 12 weeks of gestation; measured weight within the 2 weeks before delivery; and a documented height. **Exclusion criteria**: Women whose pregnancies were affected by fetal chromosomal abnormalities; only the first pregnancy was included if more than one pregnancies within the study period.	**Adjusted for:** Gestational age, maternal age, Medicaid status, parity, pre-pregnancy BMI, race/ethnicity, smoking**. Excluded:** Women with diabetes (both chronic and gestational) or hypertensive disorders (either chronic or gestational hypertension).	**Primary**: SGA, LGA. **Secondary**: Macrosomia.

^a^ All had a retrospective cohort study design

Abbreviations: ACOG = American College of Obstetricians and Gynecologists, BMI = body mass index, GDM = gestational diabetes mellitus, LBW = low birth weight, LGA = large for gestational age, NICU = neonatal intensive care unit, NR = not reported, SGA = small for gestational age, USA = United States of America, WIC = Women, Infants, and Children.

### Quality Score

Five studies achieved a score of five out of a maximum of seven on the modified Newcastle-Ottawa Scale (Table 2), while one study scored only two points [33]. Four studies had a representative sample of the pregnant population in the study setting, and two studies only included low-income populations [33,38]. The comparative groups of exposed (those with GWL) and non-exposed (GWG within guidelines) were both sampled from the same population in all studies. In five studies, GWG/GWL data were taken from medical records [23,34–36,38], whereas one study presented self-reported outcomes [33]. In terms of comparability, only one study received the maximum of two points [38], while the remaining five studies scored only one point [23,33–36]. In one study, outcomes were self-reported [33]. All studies had greater than 10% loss to follow up and two studies were judged as underpowered [36,38].

**Table 2:** Quality assessment using the modified Newcastle-Ottawa scale of included cohort studies in systematic review of gestational weight loss in obese women and adverse pregnancy outcomes.

**Study ID**	Modified New-castle Ottawa Scale							
	Selection			Comparability^a^	Outcome			
	Representativeness of exposed cohort (Maximum:★)	Selection of non-exposed cohort (Maximum:★)	Ascertainment of exposure (Maximum:★)	Comparability of cohorts on the basis of the design or analysis (Maximum:★★)	Assessment of outcome (Maximum:★)	Adequacy of follow up of cohorts (Maximum:★)	**Total score (out of 7)**	**Power**
Blomberg 2011 [23]	★	★	★	SGA ★★; LGA–★	★	–	★★★★★ (5)	Adequately powered
Durie 2011 [34]	★	★	★	SGA ★★; LGA–★	★	–	★★★★★ (5)	Adequately powered
Hinkle 2010 [33]	–	★	–	SGA–★; LGA–★	–	–	★★ (2)	Adequately powered
Kominiarek 2013 [36]	★	★	★	SGA ★★; LGA–★	★	–	★★★★★ (5)	Underpowered
Park 2011 [35]	★	★	★	SGA ★★; LGA–★	★	–	★★★★★ (5)	Adequately powered
Vesco 2011 [38]	–	★	★	SGA ★★; LGA ★★	★	–	★★★★★(5)	Underpowered

^a^ Scores were allocated for primary outcomes as follows. For SGA, one point was allocated if the study adjusted for parity, with an additional point given if adjusted for age, smoking or chronic diabetes. For LGA and macrosomia, a point was allocated for adjusting for gestational diabetes, and an additional point was given for adjusting for age, parity, smoking, or chronic diabetes. For preterm birth, one point was allocated for adjusting for parity, and an additional point was given for adjusting for age, smoking or chronic diabetes. For PPWR, one point was allocated if the study adjusted for pre-pregnancy body mass index and an additional point for adjusting for socioeconomic status or smoking. For all other secondary outcomes, the lowest score for the primary outcomes (SGA, LGA and preterm birth) was designated. The total score was derived using the minimum score allocated for confounders.

### Outcomes

#### Primary Outcomes

None of the included studies assessed the association between GWL and preterm birth.

**Fig 2:**
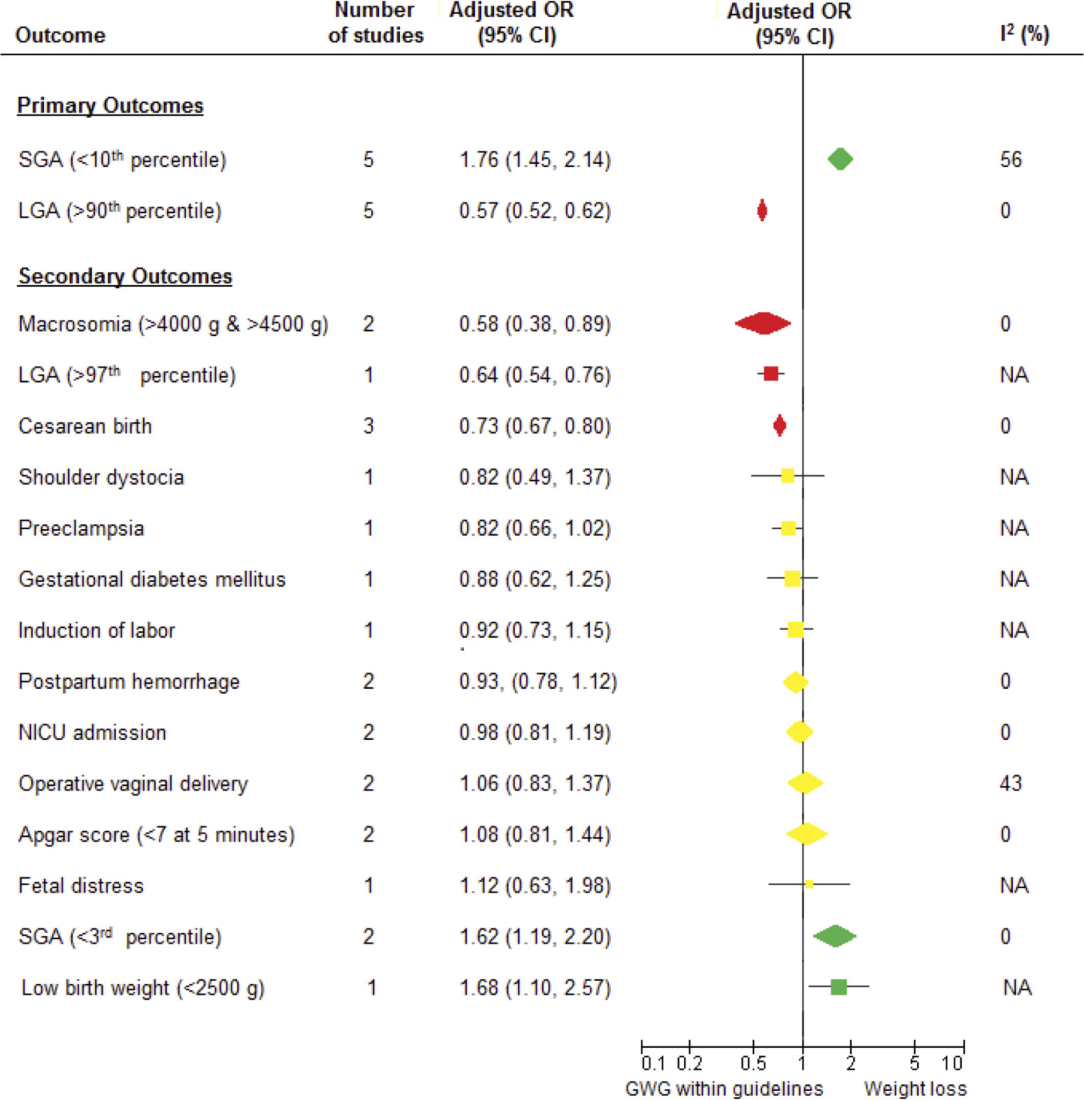
Summary of multivariable pooled odds ratios (95% confidence intervals) for the association between gestational weight loss and adverse pregnancy outcomes in obese women, compared to gestational weight gain within the 2009 Institute of Medicine guideline.

**Fig 3:**
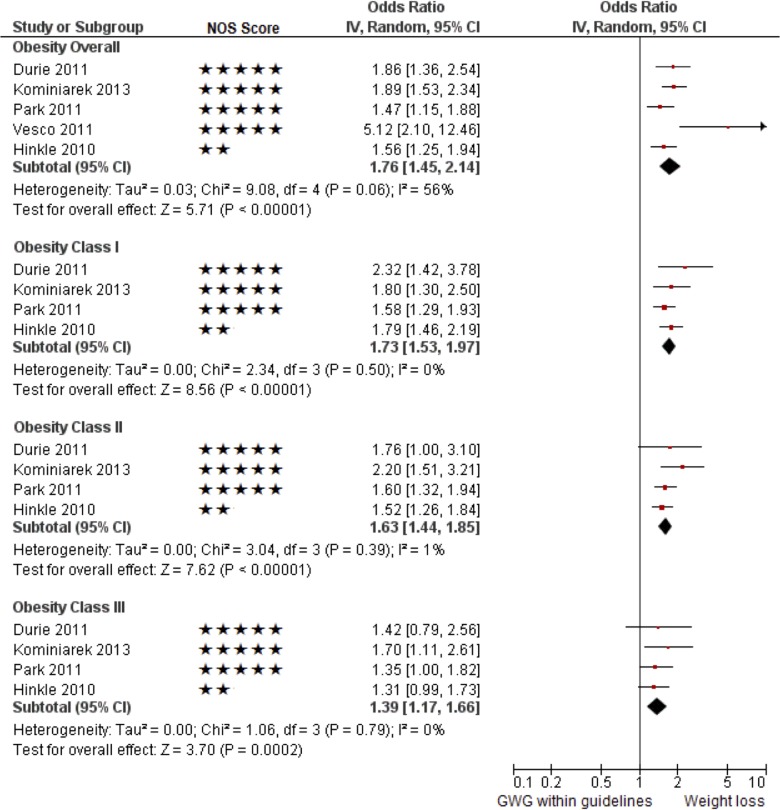
Pooled results of the studies that reported the odds of infants being small for gestational age (<10^th^ percentile, multivariable) for obese mothers with gestational weight loss compared to gestational weight gain within the 2009 Institute of Medicine guideline.

Compared to women who had GWG within guidelines, women with GWL had higher odds of SGA <10^th^ percentile (adjusted odds ratio [AOR] 1.76; 95% CI 1.45–2.14; I^2^ = 56%; five studies; Table 3, and Figs 2 and 3). The odds of SGA <10^th^ percentile decreased over the three obesity classes with a non-significant trend towards a graded relationship (for classes I, II and III, respectively, AOR 1.73; 95% CI 1.53–1.97; I^2^ = 0%; four studies; AOR 1.63; 95% CI 1.44–1.85; I^2^ = 1%; four studies and AOR 1.39; 95% CI 1.17–1.66, I^2^ = 0%; four studies). Therefore, we observed decreased odds of SGA with increasing BMI, but this relationship was not statistically significant.

**Table 3:** Summary of primary and secondary outcomes in systematic review of gestational weight loss in obese women and adverse pregnancy outcomes.

Outcome		Pooled Univariate OR			Pooled Multivariable OR		
	Obesity Classes	Number of studies	OR (95% CI)	I^2^ value (%)	Number of studies	OR (95% CI)	I^2^ value (%)
SGA (<10^th^ percentile)	**All classes**	**2**	**2.73 (1.07, 6.94)**	**79**	**5**	**1.76 (1.45, 2.14)** ^a^	**56**
	Class I	1	1.94 (1.43, 2.64)	NA	4	1.73 (1.53, 1.97) ^a^	0
	Class II	1	2.17 (1.52, 3.12)	NA	4	1.63 (1.44, 1.85) ^a^	1
	Class III	1	1.76 (1.18, 2.62)	NA	4	1.39 (1.17, 1.66) ^a^	0
LGA (>90th percentile)	**All classes**	**2**	**0.57 (0.44, 0.75)**	**0**	**5**	**0.57 (0.52, 0.62)** ^a^	**0**
	Class I	1	0.53 (0.30, 0.95)	NA	4	0.58 (0.43, 0.77) ^a^	52
	Class II	1	0.48 (0.28, 0.81)	NA	4	0.57 (0.50, 0.65) ^a^	0
	Class III	1	0.51 (0.33, 0.78)	NA	4	0.55 (0.49, 0.61) ^a^	0
Macrosomia ^b^	**All classes**	**1**	**0.65 (0.33, 1.29)**	**NA**	**2**	**0.58 (0.38, 0.89)**	**0**
	Class I	1	0.66 (0.15, 2.92)	NA	2	0.61 (0.38, 1.00)	0
	Class II	1	0.22 (0.03, 1.69)	NA	2	0.30 (0.17, 0.50)	0
	Class III	1	0.54 (0.23, 1.30)	NA	2	0.46 (0.33, 0.63)	0
LGA (>97^th^ percentile)	**All classes**	**1**	**0.74 (0.63, 0.86)**	**NA**	**1**	**0.64 (0.54, 0.76)**	**NA**
	Class I	1	0.74 (0.59, 0.93)	NA	1	0.73 (0.58, 0.92)	NA
	Class II	1	0.55 (0.41, 0.73)	NA	1	0.54 (0.40, 0.72)	NA
	Class III	1	0.68 (0.49, 0.94)	NA	1	0.64 (0.46, 0.90)	NA
Cesarean delivery	**All classes**	**2**	**0.81 (0.74, 0.88)**	**0**	**3**	**0.73 (0.67, 0.80)** ^a^	**0**
	Class I	2	0.65 (0.44, 0.95)	84	3	0.75 (0.65, 0.87) ^a^	0
	Class II	2	0.69 (0.57, 0.84)	34	3	0.73 (0.63, 0.85) ^a^	0
	Class III	2	0.80 (0.68, 0.95)	0	3	0.77 (0.66, 0.91) ^a^	0
Shoulder dystocia	**All classes**	**1**	**0.77 (0.50, 1.18)**	**NA**	**1**	**0.82 (0.49, 1.37)**	**NA**
	Class I	1	0.55 (0.29, 1.05)	NA	1	0.60 (0.32, 1.13)	NA
	Class II	1	1.26 (0.62, 2.53)	NA	1	1.30 (0.64, 2.62)	NA
	Class III	1	0.69 (0.24, 2.00)	NA	1	0.69 (0.24, 1.99)	NA
Pre-eclampsia	**All classes**	**1**	**0.89 (0.74, 1.07)**	**NA**	**1**	**0.82 (0.66, 1.02)**	**NA**
	Class I	1	0.69 (0.50, 0.94)	NA	1	0.73 (0.54, 0.99)	NA
	Class II	1	0.88 (0.65, 1.19)	NA	1	1.01 (0.74, 1.38)	NA
	Class III	1	0.69 (0.48, 0.99)	NA	1	0.74 (0.51, 1.08)	NA
Gestational diabetes mellitus	**All classes**	**-**	**-**	**-**	**1**	**0.88 (0.62, 1.25)** ^a^	**NA**
	Class I	-	-	-	1	0.97 (0.49, 1.92) ^a^	NA
	Class II	-	-	-	1	1.04 (0.56, 1.95) ^a^	NA
	Class III	-	-	-	1	0.72 (0.41, 1.26) ^a^	NA
Induction of labor	**All classes**	**-**	**-**	**-**	**1**	**0.92 (0.73, 1.15)** ^a^	**NA**
	Class I	-	-	-	1	0.90 (0.60, 1.35) ^a^	NA
	Class II	-	-	-	1	0.83 (0.55, 1.25)v	NA
	Class III	-	-	-	1	1.01 (0.70, 1.46) ^a^	NA
Postpartum hemorrhage ^c^	**All classes**	**2**	**0.92, (0.77, 1.10)**	**0**	**2**	**0.93, (0.78, 1.12)**	**0**
	Class I	2	0.84 (0.65, 1.09)	0	2	0.84 (0.65, 1.09)	0
	Class II	2	0.91 (0.47, 1.76)	54	2	0.89 (0.43, 1.86)	61
	Class III	2	0.98 (0.66, 1.47)	0	2	1.00 (0.66, 1.51)	0
NICU admission	**All classes**	**1**	**1.08 (0.86, 1.36)**	**NA**	**2**	**0.98 (0.81, 1.19)** ^a^	**0**
	Class I	1	1.13 (0.76, 1.68)	NA	2	1.12 (0.80, 1.55) ^a^	0
	Class II	1	1.01 (0.65, 1.59)	NA	2	1.02 (0.70, 1.48) ^a^	0
	Class III	1	0.82 (0.57, 1.19)	NA	2	0.86 (0.63, 1.16) ^a^	0
Operative vaginal delivery	**All classes**	**2**	**1.04 (0.82, 1.31)**	**41**	**2**	**1.06 (0.83, 1.37)**	**43**
	Class I	2	1.20 (0.80, 1.80)	57	2	1.17 (0.81, 1.68)	43
	Class II	2	0.92 (0.66, 1.27)	0	2	0.92 (0.65, 1.31)	1
	Class III	2	0.91 (0.62, 1.33)	0	2	0.98 (0.66, 1.45)	0
APGAR score (<7 at 5 minutes)	**All classes**	**2**	**1.35 (0.74, 2.43)**	**60**	**2**	**1.08 (0.81, 1.44)**	**0**
	Class I	2	1.18 (0.79, 1.75)	0	2	1.15 (0.74, 1.78)	3
	Class II	2	1.96 (0.80, 4.79)	50	2	0.99 (0.56, 1.77)	0
	Class III	2	1.03 (0.58, 1.85)	0	2	1.02 (0.57, 1.82)	0
Fetal distress	**All classes**	**1**	**0.93 (0.79, 1.09)**	**NA**	**1**	**1.12 (0.63, 1.98)**	**NA**
	Class I	1	1.01 (0.81, 1.26)	NA	1	1.84 (0.83, 4.07)	NA
	Class II	1	0.91 (0.67, 1.23)	NA	1	1.04 (0.76, 1.42)	NA
	Class III	1	0.65 (0.43, 0.99)	NA	1	0.68 (0.44, 1.05)	NA
SGA (<3^rd^ percentile)	All classes	**1**	**1.70 (1.32, 2.19)**	**NA**	**2**	**1.62 (1.19, 2.20)**	**0**
	Class I	1	2.11 (1.53, 2.91)	NA	2	2.11 (1.62, 2.76)	0
	Class II	1	0.82 (0.45, 1.50)	NA	2	1.25 (0.97, 1.60)	0
	Class III	1	2.21 (1.14, 4.29)	NA	2	1.61 (0.86, 2.99)	53
Low birth weight (<2500 g)	**All classes**	**1**	**1.67 (1.14, 2.45)**	**NA**	**1**	**1.68 (1.10, 2.57)**	**NA**
	Class I	1	1.87 (1.06, 3.30)	NA	1	1.60 (0.85, 3.01)	NA
	Class II	1	2.51 (1.26, 5.00)	NA	1	2.40 (1.14, 5.07)	NA
	Class III	1	1.11 (0.47, 2.67)	NA	1	1.10 (0.44, 2.74)	NA

Abbreviations: APGAR = Appearance, Pulse, Grimace, Activity, Respiration, CI = confidence interval; kg = kilograms, LGA = large for gestational age, OR = odds ratio, NA = not applicable, NICU = neonatal intensive care unit, NR = not reported, SGA = small for gestational age.

^a^ Analysis included which used 99% confidence intervals by Durie 2011 [34].

^b^ For univariate analyses, definition included >4000 g. For multivariable analyses, definition included both >4000 g and >4500 g (Kominiarek 2013 [36]; Hinkle 2010 [33]).

^c^ For both univariate and multivariable analyses, definition included having bled >1000 mL (Blomberg 2011 [23]), or was undefined (Kominiarek 2013 [36]).

**Fig 4:**
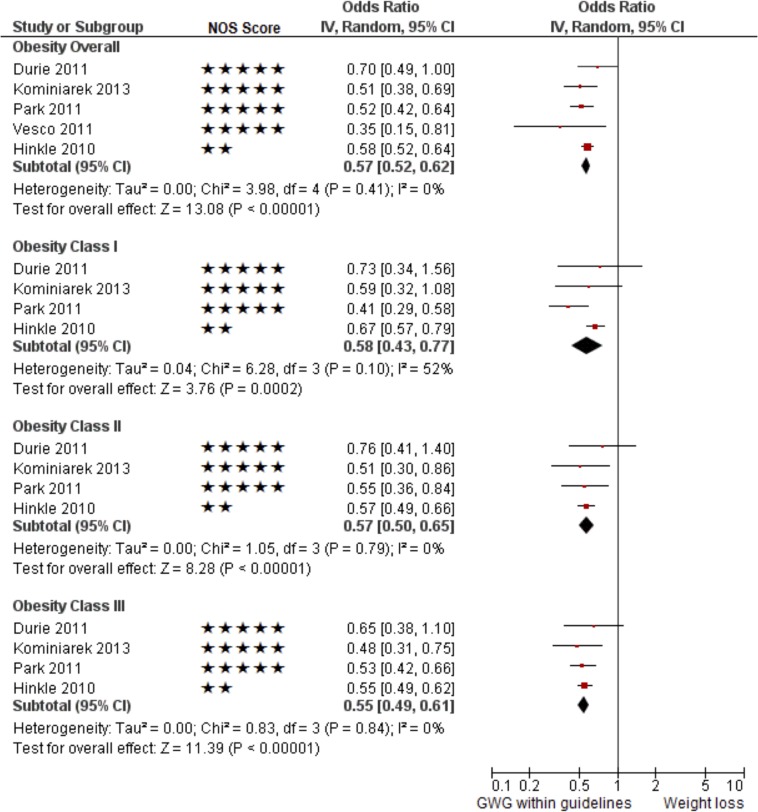
Pooled results of the studies that reported the odds of infants being large for gestational age (>90^th^ percentile, multivariable) for obese mothers with gestational weight loss compared to gestational weight gain within the 2009 Institute of Medicine guideline.

GWL was associated with a smaller odds of LGA >90^th^ percentile compared to GWG within the guidelines (AOR 0.57; 95% CI 0.52–0.62; I^2^ = 0%; five studies; Table 3, and Figs 2 and 4). The odds of LGA >90^th^ percentile was similarly reduced across each of the three obesity classes (AOR 0.58; 95% CI 0.43–0.77; I^2^ = 52%, four studies; AOR 0.57; 95% CI 0.50–0.65; I^2^ = 0%; four studies and AOR 0.55; 95% CI 0.49–0.61; I^2^ = 0%; four studies, respectively, for class I, II and III).

#### Secondary Outcomes

Significantly increased odds of SGA <3^rd^ percentile were observed for GWL in obesity overall and obesity class I (AOR 1.62; CI 1.19–2.20; I^2^ 0%; three studies; AOR 2.11; CI 1.62–2.76; I^2^ 0%; two studies, respectively). In single studies, there were significantly increased odds of low birth weight for obesity overall and class II (AOR 1.68; CI 1.10–2.57; AOR 2.40; 95% CI 1.14–5.07, respectively), and significantly decreased odds of LGA >97^th^ percentile in obesity overall and across each obesity class (AOR 0.64; CI 0.54–0.76; AOR 0.73; 95% CI 0.58–0.92; AOR 0.54; 95% CI 0.40–0.72; and AOR 0.64; 95% CI 0.46–0.90, respectively, for obesity overall, and class I, II and III). Significantly decreased odds of macrosomia were observed for GWL in obesity overall and classes II and III (AOR 0.58; CI 0.38–0.89; AOR 0.30; 95% CI 0.17–0. 50 and AOR 0.46; 95% CI 0.33–0.63, respectively; I^2^ 0% and two studies in each meta-analysis). Significantly decreased odds of cesarean birth were observed for GWL in obesity overall and across each obesity class (AOR 0.73; CI 0.67–0.80; I^2^ 0%; two studies; AOR 0.75; 95% CI 0.65–0.87; AOR 0.73; 95% CI 0.63–0.85; and AOR 0.77; 95% CI 0.66–0.91, respectively, for obesity overall, and class I, II and III). No significant association was noted for pooled AORs for postpartum hemorrhage, NICU admission, operative vaginal delivery, Apgar score <7 at 5 minutes in obese women with GWL compared to GWG within the guidelines. Pooled analyses for secondary outcomes are presented in S3–S13 Figs.

Numerous secondary outcomes remained unexplored in the included studies, such as chorioamnionitis, premature rupture of membranes, cephalo-pelvic disproportion, very low or extremely low birth weight, severe neonatal morbidity, perinatal mortality and postpartum weight retention, among others.

#### *Post hoc* Sensitivity Analysis

One study that reported 99% CIs [34] was excluded and the magnitude of the pooled estimates did not significantly change for SGA (AOR 1.76; 95% CI 1.38–2.25; I^2^ 66%; four studies) and LGA (AOR 0.56; 95% CI 0.51–0.61; I^2^ 0%; four studies).

#### Publication Bias

**Fig 5:**
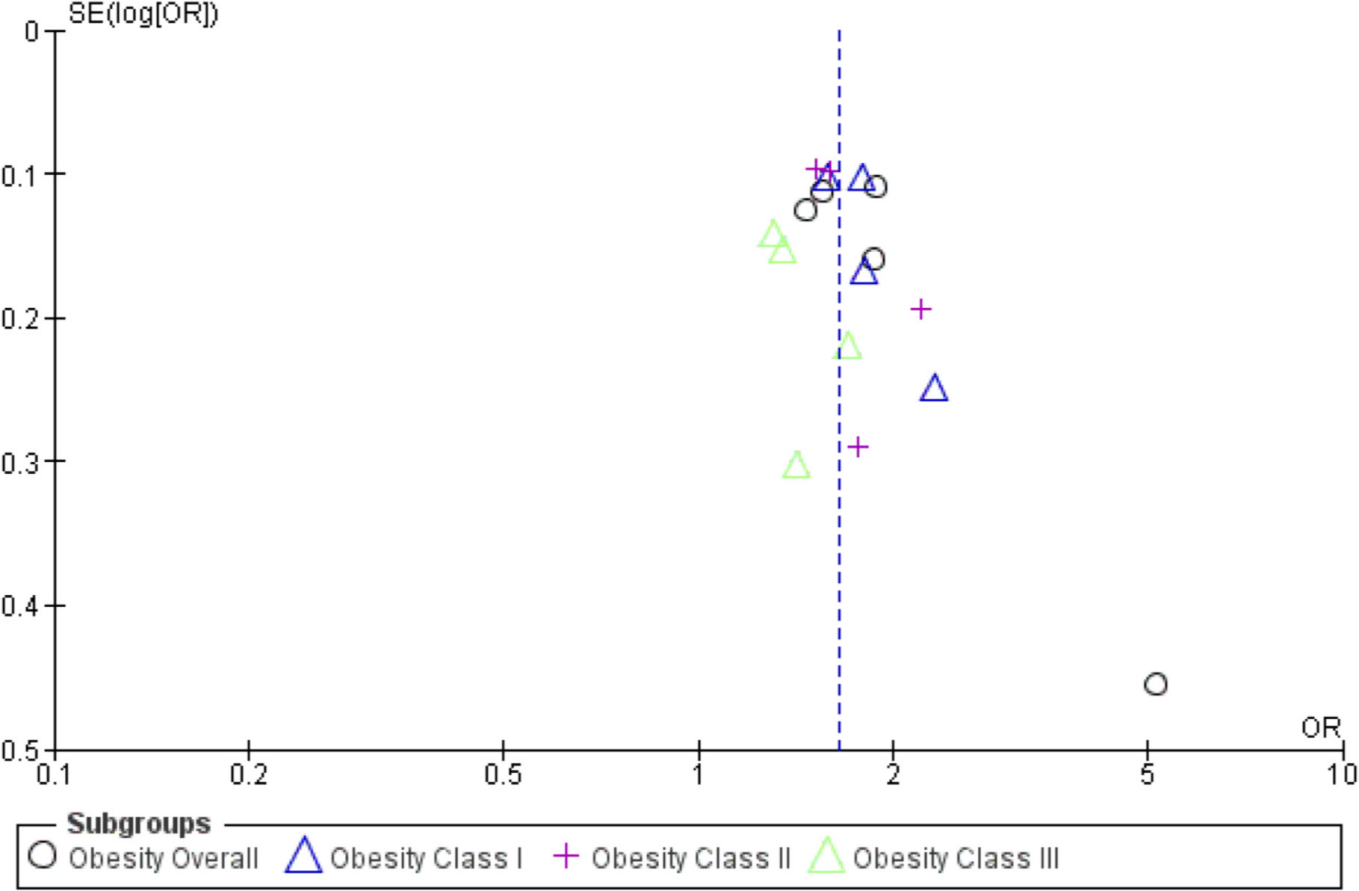
Funnel plots for effect of weight loss with SGA (<10^th^ percentile multivariable), in a systematic review of gestational weight loss in obese women and adverse pregnancy outcomes.

**Fig 6:**
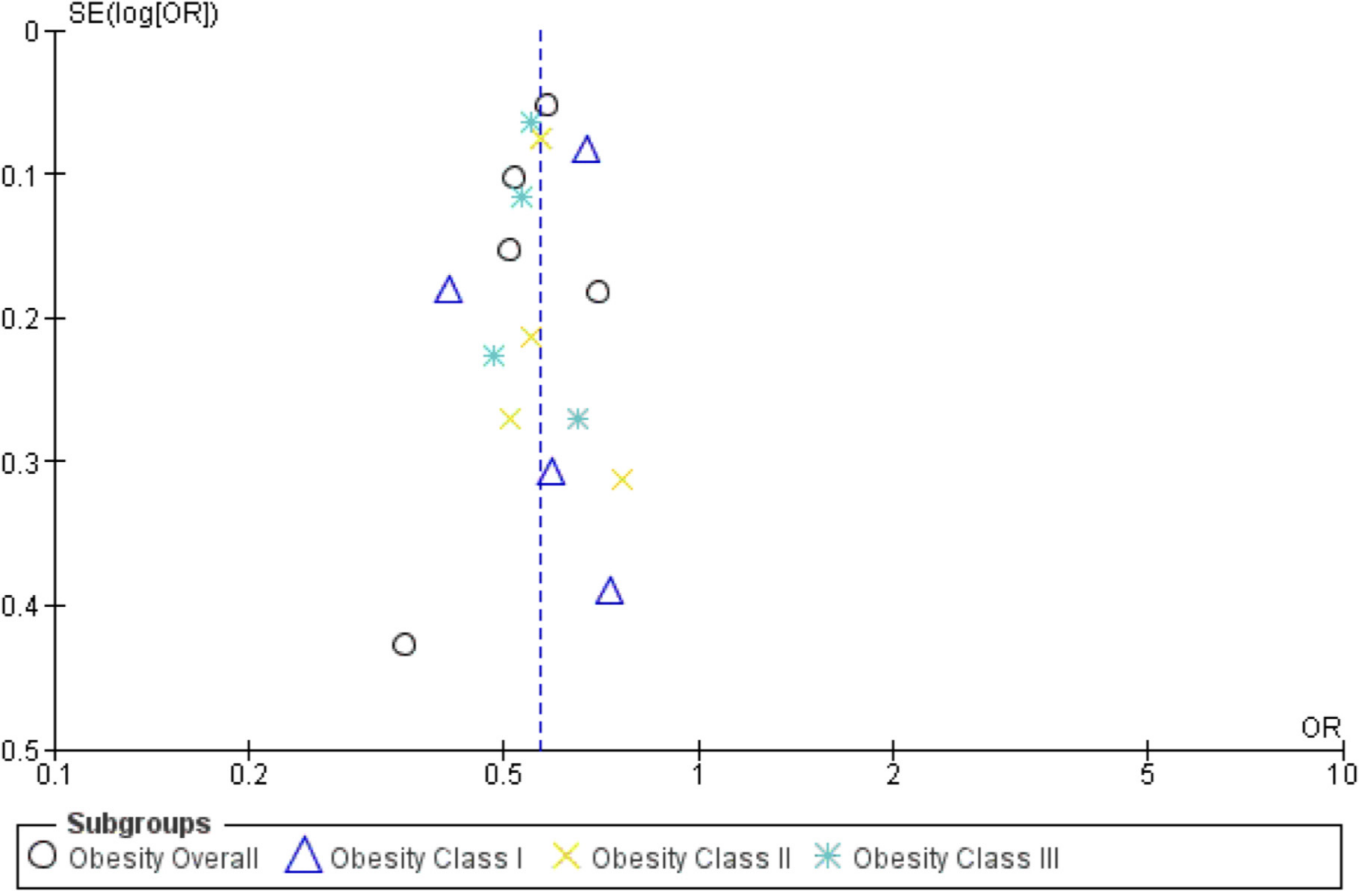
Funnel plots for effect of weight loss with LGA (>90^th^ percentile multivariable), in a systematic review of gestational weight loss in obese women and adverse pregnancy outcomes.

There was some asymmetry in the funnel plot for adjusted SGA <10^th^ percentile, suggesting potential publication bias with a possible lack of a study at the left hand side of the base of the funnel plot showing negative association (Fig 5). There was also some asymmetry in the funnel plot for adjusted LGA >90^th^ percentile suggesting potential publication bias with a possible lack of a study at the right hand side of the base of the funnel plot showing a positive association (Fig 6).

## Discussion

To the best of our knowledge this is the first systematic review to synthesize all available evidence on GWL compared to GWG within the guidelines and adverse pregnancy outcomes in obese women since the release of the 2009 IOM guidelines, which revised the recommendations for GWG in obese women. GWL was associated with an approximate doubling of odds of SGA <10^th^ percentile in obese women but an approximate halving of the odds of LGA >90^th^ percentile. Preterm birth, our other primary outcome, was not assessed in any of the included studies. GWL was associated with an increased odds of SGA <3^rd^ percentile and low birth weight but a reduction in odds of LGA >97^th^ percentile, macrosomia, cesarean birth and shoulder dystocia.

The 2009 IOM guidelines recommend specific ranges of weight gain, but not loss, in an attempt to balance the risks of preterm birth, SGA, LGA, childhood obesity, cesarean birth and postpartum weight retention. Although there were no studies examining preterm birth which met our inclusion criteria, a large retrospective cohort study did not find a significant association between preterm birth and a combined exposure of GWG below the guidelines (but still positive gain) or weight loss compared to GWG within the guidelines for the three obesity classes (AOR 1.17, 95% CI 0.94–1.46; AOR 1.14, 95% CI 0.88–1.47; AOR 1.05, 95% CI 0.80–1.38, respectively) [39]. However, our previous meta-analysis of weight gain below the guidelines (but still positive), found increased odds of preterm birth (Adjusted OR 1.46; 95% CI 1.07–2.00) [20].

The 2009 IOM guidelines acknowledged the ambiguity of literature on preterm birth due to limited epidemiologic evidence, a U-shape relationship between GWG and preterm birth that is modest in magnitude, and has uncertainty about biologic mechanisms [18].

Our findings are supported by other literature that did not meet our inclusion criteria, that indicates a trend towards a graded relationship between decreasing odds of SGA (<10^th^ percentile) with increasing obesity class. Kiel and colleagues noted that across the obesity classes, as weight gain decreased, so did the risk of four outcomes examined simultaneously, namely SGA, LGA, preeclampsia and cesarean birth, with an optimal weight gain for obesity class I of 4.5 kg to 11.3 kg, for obesity class II of a weight gain of 0 kg to 4.1 kg and for obesity class III of *loss* of 0 kg to 4.1 kg [40]. Another study recommended a weight loss of 7.6 kg for obese women to optimize SGA, LGA, preterm birth, postpartum weight retention, and childhood obesity, regardless of whether different or equal weights were given to these outcomes [15]. Our findings in this meta-analyses (adjusted OR 1.76; 95% confidence interval [CI] 1.45–2.14) are also in line with the findings in our recent meta-analysis of weight gain below the guidelines (no weight loss) was associated with an adjusted OR of 1.24 95% CI of 1.13–1.36) suggesting a graded relationship between maternal weight gain (or loss) and infant size [20].

Our systematic review determined that the odds of LGA and macrosomic infants are significantly lower in obese women with GWL compared to GWG within the guidelines. This finding is particularly important since LGA and macrosomic infants have higher risks of neonatal hypoglycemia [41], birth trauma [42] and long-term obesity [43]. Moreover, an intergenerational cycle of obesity may develop as macrosomic daughters are more likely to become obese themselves and deliver large neonates [44]. Additional research is needed to assess whether those increased risks were limited to infants in the upper 97^th^ percentile of birth weight or whether infants in the upper 90^th^-97^th^ percentile of birth weight also have similar increased risk. Weight loss in obese women was associated with a lower odds of LGA >90^th^ percentile (Adjusted OR 0.57; 95% CI 0.52–0.62) than weight gain below the guidelines but still above 0 that was noted in a recent meta-analysis (adjusted OR 0.77; 95% CI 0.73–0.81) [20].

Although there are no randomised control trials of the impact of GWL on perinatal and maternal outcomes, one situation during pregnancy in which substantial GWL occurs not infrequently is shortly after bariatric surgery. A previous systematic review reported a lower incidence of gestational diabetes, pregnancy-induced hypertension, pre-eclampsia, cesarean birth, macrosomia, and low birth weight babies in obese women following gastric banding compared to obese women without gastric banding [45] but an inconclusive association with preterm birth [45]. A large Swedish cohort study that found that women with a history of bariatric surgery had increased risks of preterm birth and SGA but a decreased risk of LGA compared to controls matched for maternal age, parity, early pregnancy body mass index, early pregnancy smoking status, educational level, and year of delivery [46]. However, the increased risks were confined to births of women with a pre-pregnancy BMI of <35 [46].

Our systematic review determined that the risk of maternal complications, such as cesarean birth, were lower in obese pregnant women with GWL. This is of interest since obese women tend to have significantly longer duration of labor compared to normal weight women [47], which might increase the risk of cesarean birth. None of the included studies investigated the association of GWL with the duration of labor and therefore more studies are required. No association was found between GWL and pooled estimates for postpartum hemorrhage and operative vaginal delivery, and single-study estimates for gestational hypertension, gestational diabetes mellitus and induction of labor. None of the included studies in our systematic review reported the timing of GWL. Timing of GWG is important since one study reported that the impact of weight gain from first to second trimester on fetal growth is highest and no effect was noted on fetal growth and weight gain from second to third trimester [48].

Strengths of this systematic review include the comprehensiveness of the search strategies in five databases, inclusion of a comprehensive list of relevant pregnancy outcomes, adherence to the PRISMA Statement [26], completion of quality assessment of included studies, and sensitivity analyses to confirm the findings of the meta-analysis. Importantly, we address the IOM’s [18] call for evidence for each obesity class. All included studies adjusted for multiple important confounders and all but one study were high quality.

Limitations of our systematic review involve the lack of studies from developing countries, therefore, limiting the generalizability of the findings, an important issue given that previous systematic reviews on obesity have found differing results from developed and developing countries [8]. None of the included studies distinguished between intentional (e.g. mediated through dietary, physical activity) or unintentional GWL. There were a relatively small number of studies that met our inclusion criteria. For some neonatal outcomes such as low birth weight, shoulder dystocia, and fetal distress, there was only one study assessing the association.

The available evidence suggests that the relationships between GWL and maternal and newborn outcomes are complex with increased odds of SGA and low birth weight contrasting with decreased odds for LGA, macrosomia and cesarean birth. Given the increased risk of SGA, a key predictor of neonatal morbidity [28] and mortality [49], and lack of adequate investigation of important pregnancy outcomes particularly preterm birth, weight loss should not be a recommendation for obese women in general.

## Supporting Information

S1 Table PRISMA Checklist for systematic review of gestational weight loss in obese women and adverse pregnancy outcomes. (DOC)

S1 Fig Pooled results of the studies that reported the odds of infants being small for gestational age (<10^th^ percentile, univariate). (TIF)

S2 Fig Pooled results of the studies that reported the odds of infants being large for gestational age (>90^th^ percentile, univariate). (TIF)

S3 Fig Pooled results of the studies that reported the odds of infants being small for gestational age (<3^rd^ percentile, multivariate). (TIF)

S4 Fig Pooled results of the studies that reported the odds of infants having an Apgar score <7 at 5 minutes (multivariate). (TIF)

S5 Fig Pooled results of the studies that reported the number of infants having an Apgar score <7 at 5 minutes (univariate). (TIF)

S6 Fig Pooled results of the studies that reported the odds of cesarean section (multivariate). (TIF)

S7 Fig Pooled results of the studies that reported the number of cesarean sections (univariate). (TIF)

S8 Fig Pooled results of the studies that reported the odds of infants being macrosomic (>4000 g or >4500 g, multivariate). (TIF)

S9 Fig Pooled results of the studies that reported the odds of infants being admitted to the neonatal intensive care unit (multivariate). (TIF)

S10 Fig Pooled results of the studies that reported the odds of operative vaginal delivery (multivariate). (TIF)

S11 Fig Pooled results of the studies that reported the number of operative vaginal deliveries (univariate). (TIF)

S12 Fig Pooled results of the studies that reported the odds of postpartum hemorrhage (multivariate). (TIF)

S13 Fig Pooled results of the studies that reported the number of postpartum hemorrhages (univariate). (TIF)

S1 File Search strategies for systematic review of gestational weight loss in obese women and adverse pregnancy outcomes. (DOC)
