# Supplementary figures and images for: Weight Loss Instead of Weight Gain within the Guidelines in Obese Women during Pregnancy: A Systematic Review and Meta-Analyses of Maternal and Infant Outcomes
Source: PLoS One. 2015 Jul 21;10(7):e0132650. doi: 10.1371/journal.pone.0132650 (PMC4509670; doi:10.1371/journal.pone.0132650)

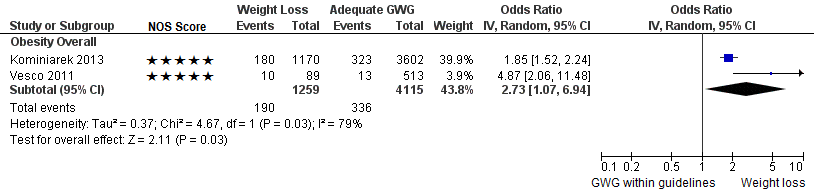

Supplement: S1 Fig — (TIF) [file pone.0132650.s002.tif]

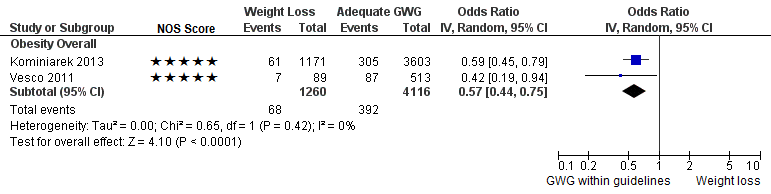

Supplement: S2 Fig — (TIF) [file pone.0132650.s003.tif]

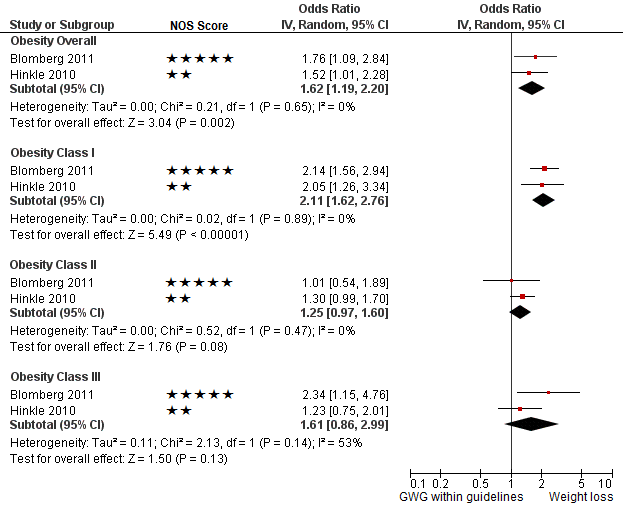

Supplement: S3 Fig — (TIF) [file pone.0132650.s004.tif]

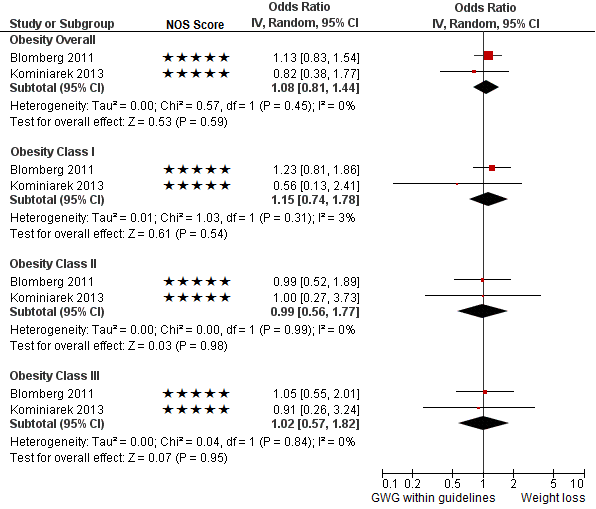

Supplement: S4 Fig — (TIF) [file pone.0132650.s005.tif]

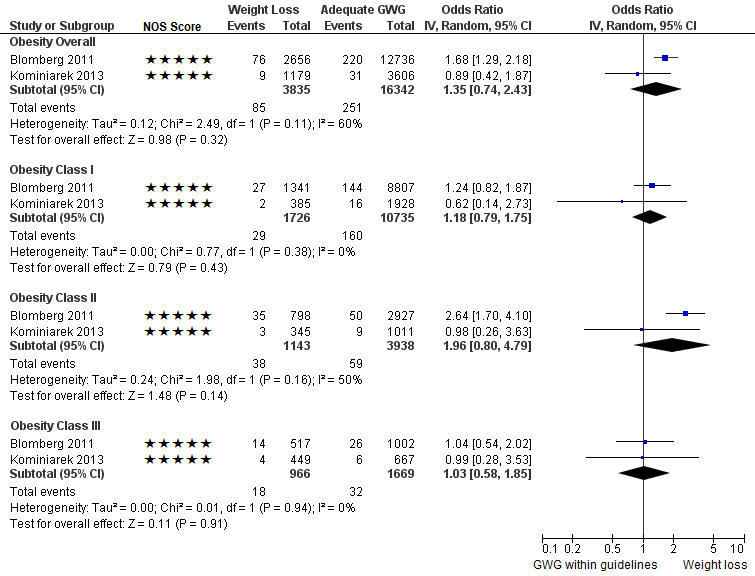

Supplement: S5 Fig — (TIF) [file pone.0132650.s006.tif]

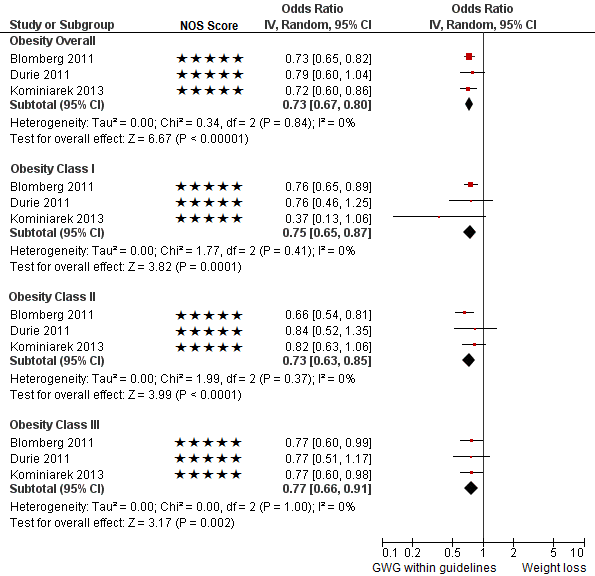

Supplement: S6 Fig — (TIF) [file pone.0132650.s007.tif]

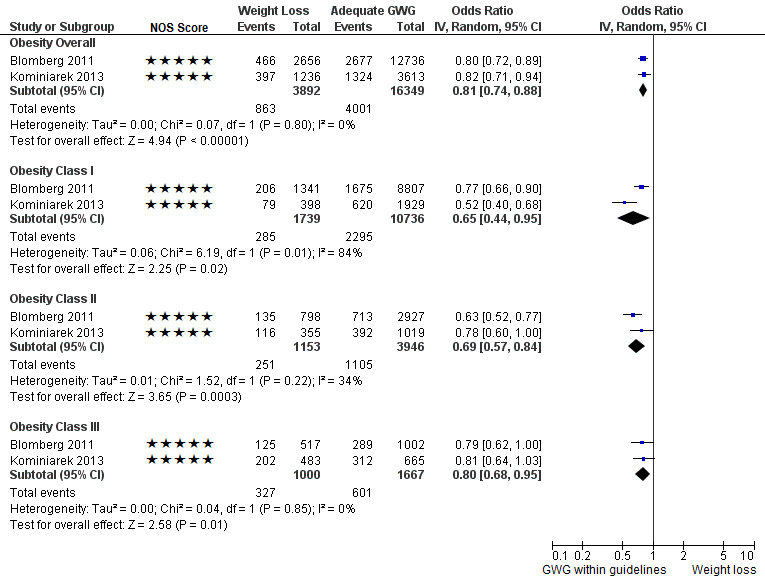

Supplement: S7 Fig — (TIF) [file pone.0132650.s008.tif]

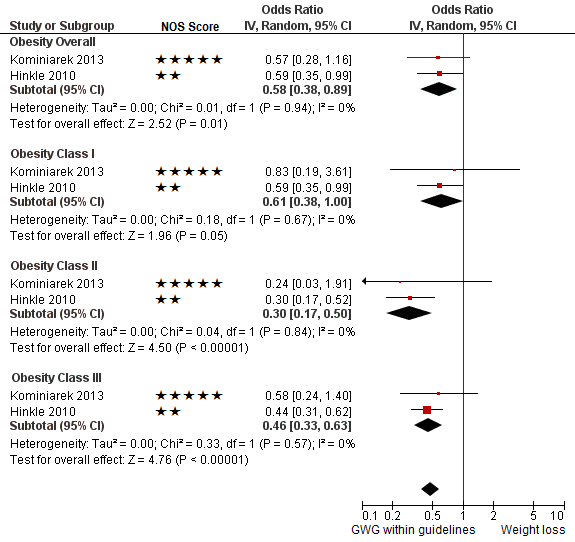

Supplement: S8 Fig — (TIF) [file pone.0132650.s009.tif]

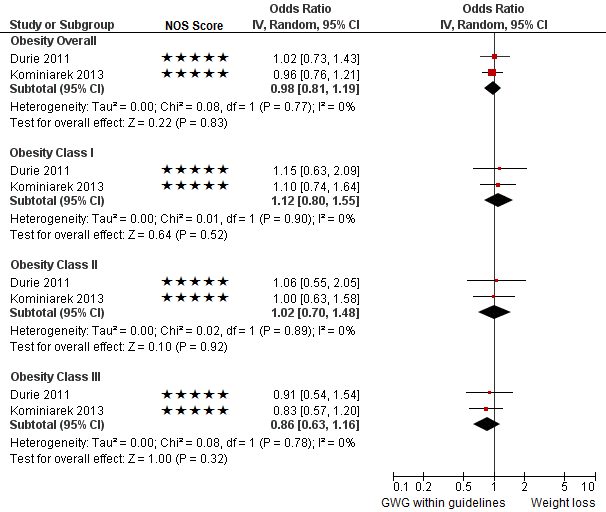

Supplement: S9 Fig — (TIF) [file pone.0132650.s010.tif]

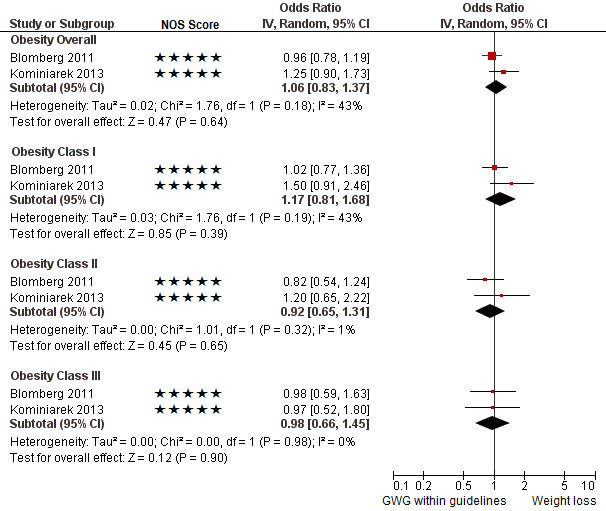

Supplement: S10 Fig — (TIF) [file pone.0132650.s011.tif]

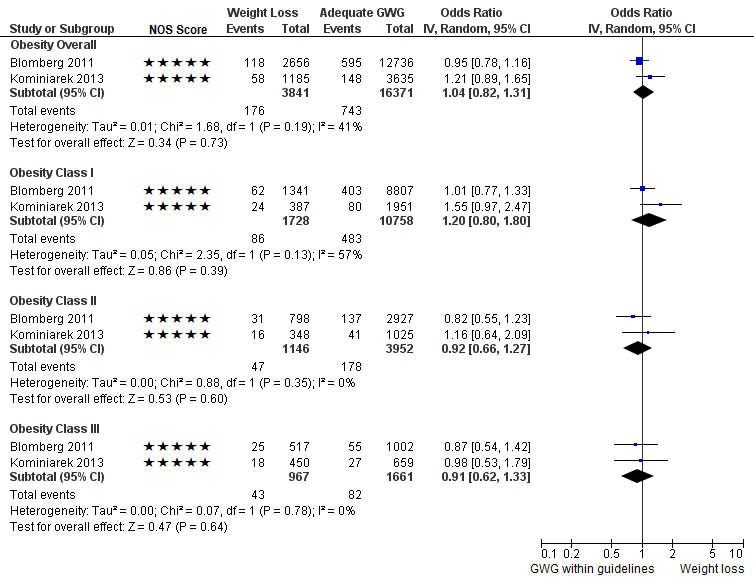

Supplement: S11 Fig — (TIF) [file pone.0132650.s012.tif]

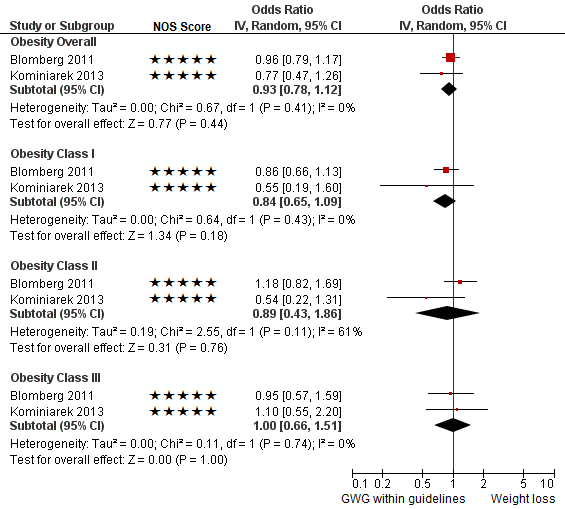

Supplement: S12 Fig — (TIF) [file pone.0132650.s013.tif]

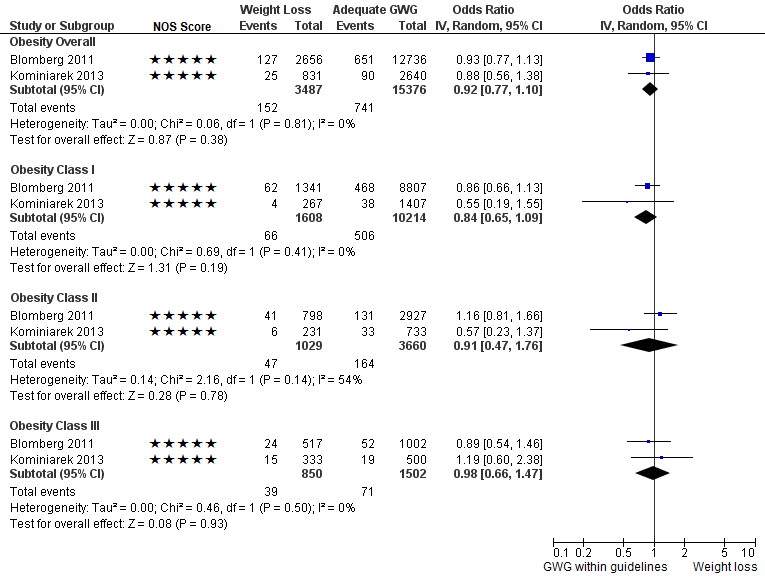

Supplement: S13 Fig — (TIF) [file pone.0132650.s014.tif]
