# Supplementary material for: Weight Loss Instead of Weight Gain within the Guidelines in Obese Women during Pregnancy: A Systematic Review and Meta-Analyses of Maternal and Infant Outcomes
Source: PLoS One. 2015 Jul 21;10(7):e0132650. doi: 10.1371/journal.pone.0132650 (PMC4509670; doi:10.1371/journal.pone.0132650)
Supplement: S1 File — (DOC) [file pone.0132650.s015.doc]

**Appendix 1: Search strategies**

Database: Ovid MEDLINE(R)
Search Strategy:
---------------------------------------------------------
1 exp Pregnancy/
2 pregnan:.mp.
3 Pregnant Women/
4 gestation:.mp.
5 maternal.mp.
6 gravid:.mp.
7 or/1-6
8 Weight Gain/
9 (weight adj4 gain:).mp.
10 (weight adj4 chang:).mp.
11 (weight adj4 increas:).mp.
12 (weight adj4 loss).mp.
13 Weight Loss/
14 (weight adj4 inadequa:).mp.
15 (weight adj4 adequa:).mp.
16 or/8-15
17 7 and 16
18 Obesity/
19 Obesity, Morbid/
20 obes:.mp.
21 Body Mass Index/
22 BMI.mp.
23 body mass index.mp.
24 (weight: adj4 categor:).mp.
25 or/18-24
26 17 and 25
27 Pregnancy Outcome/
28 (pregnan: adj4 (outcome: or complication: or high risk)).mp.
29 Infant, Small for Gestational Age/
30 Fetal Growth Retardation/
31 IUGR.mp.
32 small for gestation:.mp.
33 SGA.mp.
34 (growth adj (retard: or restrict:)).mp.
35 Fetal Macrosomia/
36 macrosomia.mp.
37 large for gestation:.mp
38 LGA.mp.
39 Premature Birth/ or Infant, Premature/
40 (premature or pre-mature pre mature).mp.
41 (preterm or pre-term or pre term).mp.
42 Infant, Low Birth Weight/ or Infant, Very Low Birth Weight/ or Birth Weight/
43 (LBW or VLBW or ELBW).mp.
44 (birth weight or birthweight).mp.
45 Birth Injuries/
46 (birth adj (trauma or injur:)).mp.
47 (shoulder adj4 dystocia).mp.
48 abortion:.mp.
49 ((neonatal: or newborn or new born or perinatal) adj4 (death or mortality or morbidity)).mp.
50 (still birth or stillbirth).mp.
51 (respiratory distress syndrome or RDS).mp.
52 ((neonat: or newborn or new born) adj4 hypoglycemia).mp
53 ((neonat: or newborn or new born) adj4 resuscitat:).mp.
54 Apgar Score/ or APGAR.mp.
55 (cord adj4 PH).mp.
56 labo?r complication:.mp.
57 intraventricular h?emmorhage.mp.
58 necrotising enterocolitis.mp.
59 retrolental fibroplasia.mp.
60 neonat: retinopathy.mp.
61 ((f?etal or newborn or neonat:) adj4 (tachycardia or tachypnea)).mp.
62 (admission: or ICU or NICU or intensive care unit or hospitali?ation).mp.
63 exp Pregnancy Complications/
64 ((pregnan: or gestation: or maternal) adj4 diabetes).mp.
65 GDM.mp.
66 (pre?eclampsia or eclampsia or PIH).mp.
67 ((pregnan: or gestation: or maternal) adj4 hypertens:).mp.
68 (c?esarean or vacuum or forcep:).mp.
69 (obstruct: adj4 labo?r).mp.
70 chorioamnionitis.mp.
71 (induc: adj4 labo?r).mp.
72 ((antepartum or postpartum) adj4 h?emorrhage).mp.
73 PPH.mp.
74 ((breast adj4 feed:) or breastfeed:).mp.
75 Breast Feeding/
76 (placenta adj4 (pr?evia or abruptio: or accreta)).mp.
77 (PROM or premature rupture of membrane:).mp.
78 (postpartum adj4 obes:).mp.
79 (postpartum adj4 weight).mp.
80 PPWR.mp.
81 or/27-80
82 26 and 81
83 Animals/
84 Humans/
85 83 not 84
86 82 not 85
87 limit 86 to yr="1990 -Current"

***************************

Database: Embase
Search Strategy:
---------------------------------------------------------
1 exp pregnancy/
2 pregnan:.mp.
3 pregnant woman/
4 gestation:.mp.
5 maternal.mp.
6 expectant mother/
7 gravid:.mp.
8 or/1-7
9 weight gain/
10 (weight adj4 gain:).mp.
11 weight reduction/
12 (weight adj4 chang:).mp.
13 (weight adj4 increas:).mp.
14 (weight adj4 loss).mp.
15 (weight adj4 inadequa:).mp.
16 (weight adj4 adequa:).mp.
17 or/9-16
18 8 and 17
19 obesity/
20 obes:.mp.
21 body mass/
22 (body mass index or BMI).mp.
23 (weight: adj4 categor:).mp.
24 or/19-23
25 18 and 24
26 pregnancy outcome/ or pregnancy complication/
27 (pregnan: adj4 (outcome: or complicat: or high risk)).mp.
28 small for date infant/
29 intrauterine growth retardation/
30 (small for gestation: or SGA).mp.
31 (growth adj4 (retard: or restrict:)).mp.
32 large for gestational age/
33 macrosomia/
34 (large for gestation: or LGA).mp.
35 macrosomia.mp.
36 prematurity/
37 (premature or preterm).mp.
38 (pre mature or pre term).mp.
39 birth weight/
40 low birth weight/
41 very low birth weight/
42 extremely low birth weight/
43 (Birth weight or birthweight).mp.
44 (LBW or VLBW or ELBW).mp.
45 birth injury/
46 (birth adj4 (injur: or trauma)).mp.
47 shoulder dystocia/
48 (shoulder adj4 dystocia).mp.
49 exp perinatal morbidity/
50 ((perinatal or neonatal) adj4 morbidity).mp.
51 respiratory distress syndrome/
52 (respiratory distress syndrome or RDS).mp.
53 intraventricular h?emorrhage.mp.
54 necrotizing enterocolitis/
55 necrotizing enterocolitis.mp.
56 retrolental fibroplasia/
57 "transient tachypnea of the newborn"/
58 (neonatal retinopathy or retrolental fibroplasia).mp.
59 ((tachycardia or tachypnea) adj4 (newborn or neonat: or f?etal)).mp.
60 Apgar score/
61 Apgar.mp.
62 (cord adj4 PH).mp.
63 ((neonat: or newborn or new born) adj4 hypoglycem:).mp.
64 ((neonat: or newborn or new born) adj4 resuscitat:).mp.
65 exp perinatal mortality/ or stillbirth/ or fetus death/
66 ((neonat: or newborn or new born or perinatal) adj4 (death or mortality)).mp
67 eclampsia/
68 preeclampsia/
69 maternal hypertension/
70 (pre?eclampsia or eclampsia or PIH).mp.
71 ((pregnan: or maternal or gestation:) adj4 hypertension:).mp.
72 pregnancy diabetes mellitus/
73 ((pregnan: or maternal or gestation:) adj4 diabetes:).mp
74 premature fetus membrane rupture/
75 (premature rupture of membrane: or PROM).mp.
76 placenta disorder/
77 (placenta: adj4 (pr?evia or abruptio: or accreta)).mp.
78 chorioamnionitis.mp. or chorioamnionitis/
79 (obstruct: adj4 labo?r).mp.
80 exp instrumental delivery/
81 (c?esarean or forcep: or vacuum).mp.
82 exp obstetric hemorrhage/
83 ((antepartum or postpartum) adj4 h?emorrhage).mp.
84 PPH.mp.
85 (admission: or ICU or NICU or intensive care unit or hospital:).mp.
86 ((breast adj4 feed:) or breastfeed:).mp.
87 breast feeding/
88 ((postpartum or post partum) adj4 weight).mp.
89 PPWR.mp.
90 or/26-89
91 25 and 90
92 Animals/
93 Humans/
94 92 not 93
95 91 not 94
96 limit 95 to yr="1990 -Current"

***************************

Database: EBM Reviews - Cochrane Central Register of Controlled Trials
Search Strategy:
---------------------------------------------------------
1 exp Pregnancy/
2 pregnan:.mp.
3 Pregnant Women/
4 gestation:.mp.
5 maternal.mp.
6 gravid:.mp.
7 or/1-6
8 Weight Gain/
9 (weight adj4 gain:).mp.
10 (weight adj4 chang:).mp.
11 (weight adj4 increas:).mp.
12 (weight adj4 loss).mp.
13 Weight Loss/
14 (weight adj4 inadequa:).mp.
15 (weight adj4 adequa:).mp.
16 or/8-15
17 7 and 16
18 Obesity/
19 Obesity, Morbid/
20 obes:.mp.
21 Body Mass Index/
22 BMI.mp.
23 body mass index.mp.
24 (weight: adj4 categor:).mp.
25 or/18-24
26 17 and 25
27 Pregnancy Outcome/
28 (pregnan: adj4 (outcome: or complication: or high risk)).mp.
29 Infant, Small for Gestational Age/
30 Fetal Growth Retardation/
31 IUGR.mp.
32 small for gestation:.mp.
33 SGA.mp.
34 (growth adj (retard: or restrict:)).mp.
35 Fetal Macrosomia/
36 macrosomia.mp.
37 large for gestation:.mp.
38 LGA.mp.
39 Premature Birth/ or Infant, Premature/
40 (premature or pre-mature pre mature).mp.
41 (preterm or pre-term or pre term).mp.
42 Infant, Low Birth Weight/ or Infant, Very Low Birth Weight/ or Birth Weight/
43 (LBW or VLBW or ELBW).mp.
44 (birth weight or birthweight).mp.
45 Birth Injuries/
46 (birth adj (trauma or injur:)).mp.
47 (shoulder adj4 dystocia).mp.
48 abortion:.mp.
49 ((neonatal: or newborn or new born or perinatal) adj4 (death or mortality or morbidity)).mp.
50 (still birth or stillbirth).mp.
51 (respiratory distress syndrome or RDS).mp.
52 ((neonat: or newborn or new born) adj4 hypoglycemia).mp.
53 ((neonat: or newborn or new born) adj4 resuscitat:).mp.
54 Apgar Score/ or APGAR.mp.
55 (cord adj4 PH).mp.
56 labo?r complication:.mp.
57 intraventricular h?emmorhage.mp.
58 necrotising enterocolitis.mp.
59 retrolental fibroplasia.mp.
60 neonat: retinopathy.mp.
61 ((f?etal or newborn or neonat:) adj4 (tachycardia or tachypnea)).mp.
62 (admission: or ICU or NICU or intensive care unit or hospitali?ation).mp.
63 exp Pregnancy Complications/
64 ((pregnan: or gestation: or maternal) adj4 diabetes).mp.
65 GDM.mp.
66 (pre?eclampsia or eclampsia or PIH).mp.
67 ((pregnan: or gestation: or maternal) adj4 hypertens:).mp.
68 (c?esarean or vacuum or forcep:).mp.
69 (obstruct: adj4 labo?r).mp.
70 chorioamnionitis.mp.
71 (induc: adj4 labo?r).mp.
72 ((antepartum or postpartum) adj4 h?emorrhage).mp.
73 PPH.mp.
74 ((breast adj4 feed:) or breastfeed:).mp.
75 Breast Feeding/
76 (placenta adj4 (pr?evia or abruptio: or accreta)).mp.
77 (PROM or premature rupture of membrane:).mp.
78 (postpartum adj4 obes:).mp.
79 (postpartum adj4 weight).mp.
80 or/27-79
81 26 and 80
82 limit 81 to yr="1990 -Current"

***************************

**CINAH**L

| S23 and S61 | **Search modes** - Boolean/Phrase |
| --- | --- |
| S61 | s24 or s25 or s26 or s27 or s28 or s29 or s30 or s31 or s32 or s33 or s34 or s35 or s36 or s37 or s38 or s39 or s40 or s41 or s42 or s43 or s44 or s45 or s46 or s47 or s48 or s49 or s50 or s51 or s52 or s53 or s54 or s55 or s56 or s57 or s58 or s59 or s60 |
| S60 | "PPWR" |
| S59 | "postpartum weight" |
| S58 | AB breast N4 Feed* |
| S57 | (MM "Breast Feeding") |
| S56 | (MH "Obstetrical Forceps") |
| S55 | (MM "Vacuum Extraction, Obstetrical") OR "vacuum" |
| S54 | (MM "Cesarean Section") OR "Cesarean" |
| S53 | AB obstruct* N4 labo?r |
| S52 | (MH "Chorioamnionitis") OR "chorioamnionitis" |
| S51 | (MH "Placenta Praevia") OR (MH "Abruptio Placentae") OR (MH "Placenta Accreta") |
| S50 | AB premature rupture |
| S49 | (MM "Fetal Membranes, Premature Rupture") |
| S48 | AB gestation* and diabetes |
| S47 | (MH "Diabetes Mellitus, Gestational") |
| S46 | (MM "Eclampsia") OR "eclampsia" OR (MM "Pre-Eclampsia") OR (MM "Pregnancy-Induced Hypertension") OR (MM "HELLP Syndrome") |
| S45 | (MM "Intensive Care Units, Neonatal") |
| S44 | (MM "Apgar Score") OR "APGAR" |
| S43 | (MM "Retinopathy of Prematurity") OR "retrolental fibroplasia" |
| S42 | (MM "Enterocolitis, Necrotizing") OR "necrotising enterocolitis" |
| S41 | (MM "Meconium Aspiration") OR "meconium aspiration" |
| S40 | (MM "Respiratory Distress Syndrome") OR "respiratory distress syndrome" |
| S39 | AB ( perinatal or neonatal ) AND AB mortality |
| S38 | AB ( perinatal or neonatal ) AND AB mortality |
| S37 | AB shoulder N4 dystocia |
| S36 | (MH "Birth Injuries+") |
| S35 | AB birth N4 weight |
| S34 | (MM "Birth Weight") OR (MM "Infant, Very Low Birth Weight") OR (MM "Infant, Low Birth Weight") |
| S33 | AB prematur* or preterm* |
| S32 | (MM "Infant, Premature") |
| S31 | "LGA" |
| S30 | "large for gestational age" |
| S29 | (MH "Fetal Macrosomia") OR "macrosomia" OR (MH "Infant, Large for Gestational Age") |
| S28 | (MM "Fetal Growth Retardation") |
| S27 | AB SGA |
| S26 | AB small for gestational age |
| S25 | (MM "Infant, Small for Gestational Age") |
| S24 | (MM "Pregnancy Outcomes") |
| S23 | S16 and S22 |
| S22 | S17 or S18 or S19 or S20 or S21 |
| S21 | AB BMI |
| S20 | AB body mass index |
| S19 | (MH "Body Mass Index") |
| S18 | "obes*" or "weight* categor*" |
| S17 | (MM "Obesity") OR (MM "Obesity, Morbid") |
| S16 | S7 and S15 |
| S15 | S8 or S9 or S10 or S11 or S12 or S13 or S14 |
| S14 | AB weight N4 loss |
| S13 | (MM "Weight Loss") |
| S12 | AB weight N4 excess* |
| S11 | AB weight N4 change |
| S10 | AB weight N4 gain* |
| S9 | (MH "Weight Gain") |
| S8 | (MM "Body Weight Changes") |
| S7 | S1 or S2 or S3 or S4 or S5 or S6 |
| S6 | "maternal" |
| S5 | "gravid*" |
| S4 | "gestation*" |
| S3 | (MM "Expectant Mothers") |
| S2 | "pregnan*" |
| S1 | (MM "Pregnancy") |

Web of Knowledge

| # 41 | [**2,198**](http://apps.webofknowledge.com/summary.do?product=WOS&doc=1&qid=41&SID=3DcFi223V2ueVsWa22z&search_mode=CombineSearches) | #40 AND #15 |
| --- | --- | --- |
| # 40 | [**445,662**](http://apps.webofknowledge.com/summary.do?product=WOS&doc=1&qid=40&SID=3DcFi223V2ueVsWa22z&search_mode=CombineSearches) | #39 OR #38 OR #37 OR #36 OR #35 OR #34 OR #33 OR #32 OR #31 OR #30 OR #29 OR #28 OR #27 OR #26 OR #25 OR #24 OR #23 OR #22 OR #21 OR #20 OR #19 OR #18 OR #17 OR #16 |
| # 39 | [**780**](http://apps.webofknowledge.com/summary.do?product=WOS&doc=1&qid=39&SID=3DcFi223V2ueVsWa22z&search_mode=GeneralSearch) | Topic=(postpartum Near/4 weight) |
| # 38 | [**10,567**](http://apps.webofknowledge.com/summary.do?product=WOS&doc=1&qid=38&SID=3DcFi223V2ueVsWa22z&search_mode=GeneralSearch) | Topic=(breastfeed*) |
| # 37 | [**2,935**](http://apps.webofknowledge.com/summary.do?product=WOS&doc=1&qid=37&SID=3DcFi223V2ueVsWa22z&search_mode=GeneralSearch) | Topic=(antepartum or obstetric*) AND Topic=(hemorrhage) |
| # 36 | [**189,965**](http://apps.webofknowledge.com/summary.do?product=WOS&doc=1&qid=36&SID=3DcFi223V2ueVsWa22z&search_mode=GeneralSearch) | Topic=(c?esarean) OR Topic=(forcep*) OR Topic=(vacuum) |
| # 35 | [**2,608**](http://apps.webofknowledge.com/summary.do?product=WOS&doc=1&qid=35&SID=3DcFi223V2ueVsWa22z&search_mode=GeneralSearch) | Topic=(placenta previa) OR Topic=(placenta accreta) OR Topic=(abruptio placenta) |
| # 34 | [**4,025**](http://apps.webofknowledge.com/summary.do?product=WOS&doc=1&qid=34&SID=3DcFi223V2ueVsWa22z&search_mode=GeneralSearch) | Topic=(premature rupture of membrane*) |
| # 33 | [**7,819**](http://apps.webofknowledge.com/summary.do?product=WOS&doc=1&qid=33&SID=3DcFi223V2ueVsWa22z&search_mode=GeneralSearch) | Topic=(gestational Near/4 diabetes) |
| # 32 | [**25,459**](http://apps.webofknowledge.com/summary.do?product=WOS&doc=1&qid=32&SID=3DcFi223V2ueVsWa22z&search_mode=GeneralSearch) | Topic=(eclampsia) OR Topic=(pregnancy induced hypertension) OR Topic=(preeclampsia) |
| # 31 | [**950**](http://apps.webofknowledge.com/summary.do?product=WOS&doc=1&qid=31&SID=3DcFi223V2ueVsWa22z&search_mode=GeneralSearch) | Topic=(tachycardia or tachypnea) AND Topic=(newborn or neonatal) |
| # 30 | [**4,958**](http://apps.webofknowledge.com/summary.do?product=WOS&doc=1&qid=30&SID=3DcFi223V2ueVsWa22z&search_mode=GeneralSearch) | Topic=(APGAR) |
| # 29 | [**588**](http://apps.webofknowledge.com/summary.do?product=WOS&doc=1&qid=29&SID=3DcFi223V2ueVsWa22z&search_mode=GeneralSearch) | Topic=(retrolental Near/4 fibroplasia) OR Topic=(neonatal Near/4 retinopathy) |
| # 28 | [**5,536**](http://apps.webofknowledge.com/summary.do?product=WOS&doc=1&qid=28&SID=3DcFi223V2ueVsWa22z&search_mode=GeneralSearch) | Topic=(necroti?ing Near/4 enterocolitis) |
| # 27 | [**28,215**](http://apps.webofknowledge.com/summary.do?product=WOS&doc=1&qid=27&SID=3DcFi223V2ueVsWa22z&search_mode=GeneralSearch) | Topic=(respiratory distress syndrome) |
| # 26 | [**1,301**](http://apps.webofknowledge.com/summary.do?product=WOS&doc=1&qid=26&SID=3DcFi223V2ueVsWa22z&search_mode=GeneralSearch) | Topic=(meconium aspiration) |
| # 25 | [**4,581**](http://apps.webofknowledge.com/summary.do?product=WOS&doc=1&qid=25&SID=3DcFi223V2ueVsWa22z&search_mode=GeneralSearch) | Topic=(perinatal morbidity) |
| # 24 | [**9,041**](http://apps.webofknowledge.com/summary.do?product=WOS&doc=1&qid=24&SID=3DcFi223V2ueVsWa22z&search_mode=GeneralSearch) | Topic=(perinatal mortality) |
| # 23 | [**12,898**](http://apps.webofknowledge.com/summary.do?product=WOS&doc=1&qid=23&SID=3DcFi223V2ueVsWa22z&search_mode=GeneralSearch) | Topic=(still birth or stillbirth) |
| # 22 | [**1,272**](http://apps.webofknowledge.com/summary.do?product=WOS&doc=1&qid=22&SID=3DcFi223V2ueVsWa22z&search_mode=GeneralSearch) | Topic=(birth Near/4 injury) |
| # 21 | [**1,070**](http://apps.webofknowledge.com/summary.do?product=WOS&doc=1&qid=21&SID=3DcFi223V2ueVsWa22z&search_mode=GeneralSearch) | Topic=(shoulder Near/4 dystocia) |
| # 20 | [**24,132**](http://apps.webofknowledge.com/summary.do?product=WOS&doc=1&qid=20&SID=3DcFi223V2ueVsWa22z&search_mode=GeneralSearch) | Topic=(pregnan* Near/4 outcome) |
| # 19 | [**99,624**](http://apps.webofknowledge.com/summary.do?product=WOS&doc=1&qid=19&SID=3DcFi223V2ueVsWa22z&search_mode=GeneralSearch) | Topic=(prematur*) |
| # 18 | [**67,552**](http://apps.webofknowledge.com/summary.do?product=WOS&doc=1&qid=18&SID=3DcFi223V2ueVsWa22z&search_mode=GeneralSearch) | Topic=(birth weight) |
| # 17 | [**6,494**](http://apps.webofknowledge.com/summary.do?product=WOS&doc=1&qid=17&SID=3DcFi223V2ueVsWa22z&search_mode=GeneralSearch) | Topic=(large for gestational age) OR Topic=(LGA) OR Topic=(macrosomia) |
| # 16 | [**9,607**](http://apps.webofknowledge.com/summary.do?product=WOS&doc=1&qid=16&SID=3DcFi223V2ueVsWa22z&search_mode=GeneralSearch) | Topic=(small for gestational age) OR Topic=(SGA) |
| # 15 | [**3,110**](http://apps.webofknowledge.com/summary.do?product=WOS&doc=1&qid=15&SID=3DcFi223V2ueVsWa22z&search_mode=CombineSearches) | #14 AND #11 |
| # 14 | [**280,623**](http://apps.webofknowledge.com/summary.do?product=WOS&doc=1&qid=14&SID=3DcFi223V2ueVsWa22z&search_mode=CombineSearches) | #13 OR #12 |
| # 13 | [**130,903**](http://apps.webofknowledge.com/summary.do?product=WOS&doc=1&qid=13&SID=3DcFi223V2ueVsWa22z&search_mode=GeneralSearch) | Topic=(body mass index or BMI) |
| # 12 | [**206,642**](http://apps.webofknowledge.com/summary.do?product=WOS&doc=1&qid=12&SID=3DcFi223V2ueVsWa22z&search_mode=GeneralSearch) | Topic=(obes*) |
| # 11 | [**9,522**](http://apps.webofknowledge.com/summary.do?product=WOS&doc=1&qid=11&SID=3DcFi223V2ueVsWa22z&search_mode=CombineSearches) | #10 AND #5 |
| # 10 | [**148,406**](http://apps.webofknowledge.com/summary.do?product=WOS&doc=1&qid=10&SID=3DcFi223V2ueVsWa22z&search_mode=CombineSearches) | #9 OR #8 OR #7 OR #6 |
| # 9 | [**77,253**](http://apps.webofknowledge.com/summary.do?product=WOS&doc=1&qid=9&SID=3DcFi223V2ueVsWa22z&search_mode=GeneralSearch) | Topic=(weight Near/4 loss) |
| # 8 | [**27,204**](http://apps.webofknowledge.com/summary.do?product=WOS&doc=1&qid=8&SID=3DcFi223V2ueVsWa22z&search_mode=GeneralSearch) | Topic=(Weight Near/4 chang*) |
| # 7 | [**1,013**](http://apps.webofknowledge.com/summary.do?product=WOS&doc=1&qid=7&SID=3DcFi223V2ueVsWa22z&search_mode=GeneralSearch) | Topic=(Weight Near/4 access*) |
| # 6 | [**56,817**](http://apps.webofknowledge.com/summary.do?product=WOS&doc=1&qid=6&SID=3DcFi223V2ueVsWa22z&search_mode=AdvancedSearch) | TS=(Weight Near/4 Gain*) |
| # 5 | [**457,223**](http://apps.webofknowledge.com/summary.do?product=WOS&doc=1&qid=5&SID=3DcFi223V2ueVsWa22z&search_mode=AdvancedSearch) | #4 OR #3 OR #2 OR #1 |
| # 4 | [**8,312**](http://apps.webofknowledge.com/summary.do?product=WOS&doc=1&qid=4&SID=3DcFi223V2ueVsWa22z&search_mode=GeneralSearch) | Topic=(gravid*) |
| # 3 | [**162,023**](http://apps.webofknowledge.com/summary.do?product=WOS&doc=1&qid=3&SID=3DcFi223V2ueVsWa22z&search_mode=GeneralSearch) | Topic=(maternal) |
| # 2 | [**116,754**](http://apps.webofknowledge.com/summary.do?product=WOS&doc=1&qid=2&SID=3DcFi223V2ueVsWa22z&search_mode=GeneralSearch) | Topic=(gestation*) |
| # 1 | [**306,854**](http://apps.webofknowledge.com/summary.do?product=WOS&doc=1&qid=1&SID=3DcFi223V2ueVsWa22z&search_mode=GeneralSearch) | Topic=(pregnan*) |
